# U.S. adolescents’ attitudes toward school, social connection, media use, and mental health during the COVID-19 pandemic: Differences as a function of gender identity and school context

**DOI:** 10.1371/journal.pone.0276737

**Published:** 2022-10-27

**Authors:** Drew P. Cingel, Alexis R. Lauricella, Lauren B. Taylor, Hannah R. Stevens, Sarah M. Coyne, Ellen Wartella

**Affiliations:** 1 Department of Communication, University of California, Davis, Davis, California, United States of America; 2 Erikson Institute, Chicago, Illinois, United States of America; 3 School of Family Life, Brigham Young University, Provo, Utah, United States of America; 4 Department of Communication Studies, Northwestern University, Evanston, Illinois, United States of America; Albanian University, ALBANIA

## Abstract

The COVID-19 pandemic changed school contexts and social opportunities dramatically for adolescents around the world. Thus, certain adolescents may have been more susceptible to the stress of the pandemic as a function of differences in schooling. We present data from 1256 United States adolescents (ages 14–16) to examine how the 2020–2021 school context (in-person, hybrid, or virtual) related to feelings of school satisfaction and success, social connection, mental health, and media use. We also examine differences as a function of gender identity. Results demonstrate that school context, particularly in-person compared to virtual schooling, was related to higher school satisfaction and academic success, stronger feelings of social connection and inclusion, lower levels of anxiety and depression, and less problematic media use. Interestingly, adolescents did seem to use media as a tool to support social connection when in hybrid or virtual school contexts, but they also reported higher rates of problematic media use, thus suggesting that media use needs to be examined more carefully to understand its role as a potential protective mechanism for adolescents’ social connection and mental health. These findings provide baseline information about how schools’ responses to the COVID-19 pandemic may have created disparities among youth. These findings have implications for current school interventions.

## Introduction

The COVID-19 pandemic caused unprecedented disruptions to human life around the world. Perhaps no group experienced more disruption to normal life than children and adolescents, as schools quickly transitioned to virtual learning in attempts to slow the spread of the virus in the Spring of 2020, following the guidance of local and federal health agencies [1]. These changes and disruptions were not uniform, however. Rather, adolescents’ school context, whether in-person, virtual, or a hybrid of the two, varied from state to state, school district to school district, and even within families. Therefore, certain children and adolescents in the United States, as a function of their school context, experienced different types of disruptions in comparison to others.

It is vitally important to understand how different groups of adolescents were influenced by the pandemic in terms of their attitudes toward school, sense of social connection, media use, mental health. Understanding how individuals’ school context during the pandemic relates to different social, emotional, and mental health consequences is important for policy makers, educators, and parents, particularly so that adolescents can be supported as the re-enter school during the 2021–2022 school year and beyond. Moreover, this understanding of whether adolescents were able to utilize technology as a protective mechanism during a time of such immense change is important for how we understand adolescent media use and its correlates. Further, given noted gender differences in media use [2, 3], school experiences [4], and mental health [5], we also examine the role of gender identity in these processes.

Parents, educators, and policymakers have worried about the myriad of effects of the pandemic and virtual schooling on children and adolescents. Early evidence supports these concerns, including negative attitudes toward school stemming from difficulties with virtual schooling [6], in addition to learning loss [7], increased media consumption [8], and poor mental health [9]. Further, although rates of anxiety and depression, already growing among adolescents around the world [10], increased during the pandemic [9], a fear of a lost academic year also related to adolescents’ mental distress [7]. While there is some evidence of excessive and potentially addictive media use among adolescents during the pandemic [8], other studies among young adults show that media use may have served as an adaptive coping mechanism, offering the potential for mediated connection during a time of decreased in-person contact [11]. In all, these studies suggest that factors related to the pandemic are associated with adolescents’ perceptions of and success in school, their media use, and their mental health.

To our knowledge, however, research has not yet examined how different school contexts during the COVID-19 pandemic impacted these relationships. Therefore, the goal of the present paper is to examine data from a national sample of 1256 American adolescents (ages 14–16) one year after the start of the COVID-19 pandemic (data collected May-June 2021). We analyzed differences in adolescents’ self-reported attitudes about school, school success, social connectedness, social media and video game use, and mental health as a function of their gender identity and school context: either in-person, virtual, or a hybrid with some in-person and virtual components at the end of the 2020–2021 school year. We specifically considered social media and video game use, given their potential to increase social connection [12, 13] but also to be used problematically [14, 15].

### The diathesis-stress model and school context

A common theory applied to adolescent development and mental health during periods of stress is the diathesis-stress model. This model posits that mental health disturbances, and the trajectory of such disturbances, are the result of the interaction between individual susceptibilities and stress experienced during life [16]. Individual susceptibilities (diatheses) can include those that are genetic, psychological, biological, or situational. In the current study, we conceptualize adolescent gender identity and school context during the pandemic as two individual susceptibilities. The pandemic and resultant disruptions were certainly causes of stress to children and adolescents around the world. The model predicts, however, that certain individuals will be more likely to experience mental health disturbances based on individual factors. We argue that the disruption and stress caused by the pandemic was exacerbated among adolescents participating in virtual schooling. Indeed, initial studies demonstrated that adolescents in virtual schooling experienced various difficulties [6] and worries about learning loss [7] that were presumably not experienced to the same extent among those participating in in-person or hybrid school contexts. Thus, school context may have served as a situational difference variable among adolescents.

Although the model is typically used to predict mental disorders, it has also been used to predict other outcomes related to poor well-being, including problematic media use [17]. Given that adolescents and young adults reported numerous technological issues with participating in online schooling [6, 18], it is also likely that grades and general satisfaction with school suffered in virtual contexts. Further, with the closure of schools and the move to virtual learning due to the COVID-19 pandemic, typical routines were severely impacted, as these adolescents were forced to spend more time at home, either alone or with family members, and less time with their peers. Conversely, those attending in-person or hybrid schooling were still able, to a degree, to interact with friends and peers, while not having to deal with the potential technological issues associated with virtual schooling. Thus, in addition to academic success and school satisfaction, adolescents’ sense of connection with peers is likely to have been negatively affected by virtual schooling. Though the long-term impact of this change on adolescents may not be understood for some time, in the short-term there are likely significant harms to adolescent mental health, as well as their feelings about school and academic success, and sense of social connection. Therefore, we expect that:

H1: Adolescents in virtual school contexts will report lower satisfaction with school and lower academic success (e.g., grades) compared to adolescents who experienced hybrid or in-person education.
H2: Adolescents in virtual school contexts will report a lower sense of social connection compared to adolescents who experienced hybrid or in-person education.
H3: Adolescents in virtual school contexts will report worse mental health compared to adolescents who experienced hybrid or in-person education.

### Adolescents’ school context and media use

Media, particularly social media, have long been used by adolescents to maintain a sense of connection with their friends and peers, with positive implications for well-being [19]. The diathesis-stress model posits that certain variables can serve as protective factors, which buffer against the effect of stressors. Given that adolescents in virtual schooling are likely to have fewer opportunities to interact with friends and peers, relative to those with at least some in-person component, we expect that the use of media to promote social connections between adolescents and their peers will be higher among adolescents in virtual schooling. In this way, media may be used to fulfill a sense of social connection and inclusion that is lost when school is not in person. However, as adolescents spend more time online and depend on online communication for social connection, they may also increase their risk of developing internet addiction or other problematic uses of the Internet. Indeed, there is some evidence that peers can promote internet addiction and other risky online behaviors among adolescents [20]. Further, researchers have found that adolescents with more social support were less at risk for internet addiction [21]. Given our expectation that adolescents in virtual schooling will report lower satisfaction with social connections, we also expect that adolescents will also be more at risk for reporting problematic media use. Therefore, we predict:

H4: Adolescents in virtual school contexts will report (a) higher levels of social media use and video gaming for social connection and (b) higher levels of problematic social media use and video gaming compared to adolescents who experienced hybrid or in-person education.

### Adolescent development and gender identity

As noted, gender identity may be another vulnerability factor, as conceptualized by the diathesis-stress model, that could explain why certain adolescents may be more susceptible to negative outcomes after experiencing the stress of the pandemic. The LGBTQ+ population encompasses a variety of gender and sexual identities, including youth whose gender expressions fall visibly outside of heteronormative societal values, such as transgender and gender-nonconforming (TGNC) youth. TGNC youth are particularly susceptible to health and academic disparities [22]. Adolescents can benefit from meaningful friendships with supportive peers to help them cope with their emotions [23, 24]. Yet, the increased prevalence of discrimination, verbal and physical victimization, and rejection from peers amongst TGNC youth can inhibit their ability to form relationships, platonic or romantic, and can inhibit a sense of connection to their peers [5, 22, 25]. Furthermore, victimization can adversely impact the academic success of TGNC youth. For example, a survey of 11,447 high school students from an urban county in the Midwestern United States found that the relationship between TGNC adolescents’ gender visibility and decreased GPA rates was partially mediated by school victimization [26]. Since TGNC youths often have a diminished sense of social connection and belonging, this sense of isolation and lack of emotional connections to peers can result in their differing attitudes about school, academic success, and mental health. We thus predict the following:

H5: TGNC adolescents will report lower satisfaction with school and lower academic success (e.g., grades) when compared to their heteronormative peers.
H6: TGNC adolescents will report a lower sense of social connection than their heteronormative peers.
H7: TGNC adolescents will report worse mental health than their heteronormative peers.

### Adolescents’ school context, gender identity, and media use

Similar to our argument for increased media use among adolescents in virtual schooling, media use may also serve as a protective factor among TGNC youth. For example, LGBTQ+ TGNC youth reported lower rates of school satisfaction, school success, social connection, peer relations, and higher rates of gender-based bullying victimization and physical bullying victimization than their male and female peers before widespread physical distancing guidelines [27, 28]. Given that online peer interactions function differently from in-person interactions [29], online environments may better facilitate TGNC adolescents’ engagement in social relationships. For example, TGNC youth may be more inclined to seek community and social support online than compared to their cis-gendered peers [3]. This may be, in part, because they experience disproportionate levels of bullying by verbal and physical victimization in in-person schooling and can express themselves and navigate unwanted comments and advances online in ways in which they cannot navigate when in-person [4].

Online communities can also function as spaces for TGNC youth to seek gender-affirming emotional support from the LGBTQ+ community in ways they do not receive support from predominantly cis-gendered peers at school [30, 31]. Further, the ability for TGNC adolescents to use their chosen names is a resilience-promoting factor [32]. Online communication allows for more selective self-presentation of the gendered attributes adolescents choose to display to their peers [33, 34]. For example, students might disclose their gender identity by adding their preferred pronouns to their zoom name. Therefore,

H8: TNGN adolescents will report (a) higher levels of social media use and video gaming for social connection and (b) higher levels of problematic social media use and video gaming compared to adolescents who identify as male or female.

Overall, we have predicted main effects of school context and gender identity on a host of outcome variables. It is unclear if there will be an interaction between the two, however. It is possible that the unique combination of identifying as TGNC and being in virtual schooling could make these individuals the most susceptible to negative outcomes following the stress of the pandemic. Conversely, it is also possible that online schooling could serve as a protective factor, providing TGNC adolescents with more opportunity for selective self-presentation while also cutting down opportunities for bullying and other forms of in-person victimization. Therefore, we ask,

RQ1: Is there a school context by gender identity interaction on adolescents’ (a) school satisfaction and academic success (e.g., grades), (b) sense of social connection, (c) mental health, or (d) media use?

## Materials and methods

### Participants

Adolescents (*N* = 1256) from the United States were recruited through a Qualtrics panel to participate in an online survey between May 20, 2021 and June 23, 2021. Participants were between the ages of 14 and 16 years old. The majority of respondents were female (*n* = 813; 65%), with 28% (*n* = 349) identifying as male, and 6% (*n* = 78) TGNC. There was racial diversity among the respondents (47.9% identifying as White/Caucasian). Please see Table 1 for a complete listing of demographic information.

**Table 1:** Sociodemographic characteristics of participants.

Baseline Characteristic	Percent	*n*
Age		
14	18.4%	231
15	35.3%	443
16	46.3%	582
Grade		
9th	36.9%	464
10th	38.9%	489
11th	24.1%	303
Gender		
Female	64.7%	813
Male	27.8%	349
Transgender	1.8%	23
Non-binary or gender queer	2.7%	34
Self-described	1.7%	21
Race/Ethnicity		
Hispanic, Latino/a, or Spanish origin	42.2%	530
White	47.9%	602
Black or African American	23.6%	296
Asian or Asian American	11.4%	143
American Indian or Alaskan Native	4.7%	59
Other	12.1%	152
Self-described	6.2%	78

Note: Race and Ethnicity variables could sum to more than 100% as people could select multiple answers.

### Procedure

We worked with the survey company, Qualtrics (Qualtrics samples from traditional, actively managed, double-opt-in market research panels as well as occasionally using social media to gather respondents. Participants in Qualtrics’ panel submit an initial registration form requesting to participate in market research studies. Participants then provide information to build their profiles with standardized lists of questions. The team at Qualtrics recruits eligible participants to complete the survey), to recruit the sample after receiving approval by the institutional review board of the first author’s university. As parents had already consented to their adolescent’s inclusion on the panel, the review board waived the need for active parent consent. Adolescents were contacted online and invited to take part in a research study. They provided assent to participate prior to answering any survey questions. The survey took approximately 15–20 minutes to complete. At the end, we thanked participants for their time, and they received compensation for their participation. Their compensation is commensurate with Qualtrics policies, and involves both cash and gift cards. This is part of an ongoing longitudinal project in which these adolescents will be contacted again; in this paper, we present data collected from Wave 1.

### Measures

#### School context

We asked participants how they participated in school this month (the month during Wave 1 data collection), or if they were already on summer break, how they participated in school the last month prior to summer break. Participant response options included “in-person in the school building”, “hybrid model where some school is in-person and some is virtual/online”, “completely virtual/online”, “a different version of school (e.g., home schooling, tutoring)”, or “did not attend school at all”. For all participants, we asked how long they had been in that particular school context on a 1–7 scale anchored by ‘less than 2 weeks’ and ‘the entire school year’.

#### Gender

We asked participants how they identified. Answer options included ‘female’, ‘male’, ‘transgender’, ‘non-binary or gender queer’, and ‘prefer to self-describe’. Those identifying as transgender, non-binary or gender queer, and those who self-described were combined into a TGNC group for comparison with females and males.

#### School satisfaction and academic success

We asked participants four questions to gauge how they were *feeling about school* when completing the survey. Respondents used 1–5 Likert-like scales to answer. For example, we asked “How do you feel about school right now?” (anchored by ‘*I don’t like school at all’* and *‘I like school very much’*; *M* = 2.78, *SD* = 1.03), “How often do you feel that the school work you are assigned is meaningful and important?” (anchored by ‘*never’* and *‘almost always’*; *M* = 2.78, *SD* = .94), “How interesting are most of your classes to you right now?” (anchored by ‘*very dull’* and *‘very exciting’*; *M* = 2.43, *SD* = .98), and “How satisfied are you with your school experience right now?” (anchored by ‘*completely dissatisfied’* and *‘completely satisfied’*; *M* = 3.83, *SD* = 1.43).

We asked participants to report “What were your grades like LAST school year (2019–2020)” and “…THIS school year (2020–2021)?” on a scale where 1 = *Mostly D’s or F’s* and 7 = *Mostly A’s*. We calculated a grade difference variable by subtracting this year’s grades from last year’s grades. Thus, a positive number indicates that grades were higher in the 2019–2020 school year compared to the 2020–2021 school year (*M*_*difference*_ = .53, *SD* = 1.78).

#### Social connection and group inclusion

Adolescents reported on how *satisfied they felt with their peer connections* through six items adapted from a social connectedness measure [35]. Items were recoded so that higher numbers indicated more satisfaction (1–5 scale anchored by *‘strongly disagree’* and *‘strongly agree’*, *M* = 2.56, *SD* = .75, α = .78). Sample statements included, “I would like to have a larger circle of friends”, “I feel a lack of contact with people in my social network”, and “I feel that people in my social network often think of me.” Adolescents responded to three items designed to measure their *sense of inclusion* in a social group [adapted from 36]: “How included do you feel in this group?” “To what extent do you feel well-integrated into this group?” and “To what extent do you feel a sense of belongingness within the group?” Items were recoded so that higher numbers indicated a higher sense of inclusion (1–5 scale anchored by *‘not at all’* and *‘very much’*, *M* = 2.68, *SD* = 1.07, α = .91).

#### Mental health

We used eight items to measure participants’ *loneliness* [37]. Sample items included “I do not feel alone”, “I feel part of a group of friends”, and “I am no longer close to anyone.” Items were recoded so that higher numbers indicated more loneliness (1–5 anchored by *‘never’* and *‘always’*, *M* = 2.94, *SD* = .80, α = .83). We used five items to measure participants’ *social anxiety*, adapted from the measure of Fear of Negative Evaluation Scale [38]. Items were recoded so that higher numbers indicated more social anxiety (1–5 scale anchored by *‘never’* and *‘always’*, *M* = 3.28, *SD* = .85, α = .68). Sample items were: “I worry about what other people will think of me even when I know it doesn’t make any difference” and “If I know someone is judging me, it has little effect on me.” We used 8 of the 9-items from the Patient Health Questionnaire-9 [39] to measure *depression*. We excluded item 9 (suicidal risk) because according to the authors, a respondent who answers ‘yes’ to this item needs further, and immediate, assessment for suicidal risk by a trained professional, which we were not able to provide due to the online and national nature of the survey (1–5 scale anchored by *‘never’* and *‘always’*, *M* = 2.39, *SD* = .81 α = .88). Sample items include: “poor appetite or overeating”, “little interest or pleasure in doing things”, and “feeling down, depressed, or hopeless.” We used eight items from the Patient-Reported Outcomes Measurement Information System Short Form for Anxiety (PROMIS-SF) [40] to measure participants’ *general anxiety*. Respondents were asked to indicate how often in the past seven days they had been bothered by a list of problems on a 5-point scale. Items were recoded so that higher numbers indicated more social anxiety (1–5 scale anchored by *‘never’* and *‘always’*, *M* = 3.00, *SD* = 1.01, α = .92). Sample items include: “I felt uneasy”, “Many situations made me worry”, and “I had difficulty calming down”.

#### Social media and video game use

To measure social media use, we asked “Do you use social media?”. A majority of the sample responded ‘yes’ (*n* = 1010; 80.54%), and answered questions about social media use for social connection and problematic social media use. We used seven items to measure participants’ use of *social media for social connection*. Sample items include “Social media helped me stay connected with my friends” and “I feel disconnected from friends when I have not logged into social media” (1–5 scale anchored by *‘never’* and *‘always’*, *M* = 2.91, *SD* = .91, α = .85). We used five items to measure participants’ *problematic social media use*. Sample items include “It was hard to stop using social media” and “Social media made it harder to fall/stay asleep” (1–5 scale anchored by *‘never’* and *‘always’*, *M* = 2.41, *SD* = .91, α = .80).

To measure video game use, we asked “Do you play video games (on a computer, phone, or console)?”. Over half of the sample responded ‘yes’ (*n* = 667; 53.11%), and answered questions about video gaming for social connection and problematic video gaming. We used four items to measure participants’ use of *video gaming for social connection*. Sample items include “I felt supported by a friend while gaming” and “Gaming helped me stay connected with my friends.” (1–5 scale anchored by *‘never’* and *‘always’*, *M* = 2.71, *SD* = 1.14, α = .84). We used five items to measure participants’ *problematic video gaming*. Sample items include “It was hard to stop gaming” and “Gaming made it harder to fall/stay asleep” (1–5 scale anchored by *‘never’* and *‘always’*, *M* = 1.92, *SD* = .84, α = .79).

#### Covariates

We asked participants to report their age (either 14, 15, or 16 years). We asked participants to report the highest educational attainment of one of the parents/caregivers in the household (6-point scale ranging from *completed grade school or less* and *graduate or professional school*). Participants reported their own race/ethnicity, and for the purposes of analysis we dichotomized this variable as ‘white’ and ‘non-white’. Finally, for participants who reported being in virtual schooling, we asked how long they had been in that particular mode (7-point scale ranging from *less than 2 weeks* and *entire school year*). In order to use this variable as a control for our entire sample, we coded participants who did not ever participate in virtual schooling as a ‘0’, thus creating an eight-item scale.

## Results

### Preliminary analyses

All data were analyzed using SPSS 27. We first ran a correlation matrix with our main study variables (e.g., participant gender, school context, mental health, social connection) and possible covariates (parent education, age, ethnicity). Each covariate was significantly related with school context and variably correlated with the other key study variables. Thus, each was included as a covariate in subsequent analysis. We also controlled for the amount of time participants spent in virtual schooling throughout the school year. Next, we conducted a series of univariate (ANCOVA) and multivariate (MANCOVA) analyses of covariance with school context (in-person, virtual, hybrid) and gender (male, female, TGNC youth) as independent variables. We examined significant main effects using Bonferroni post-hoc tests. For all analyses, we report significant main effects, interactions, and post-hoc tests in the text. All other relevant information can be found in the respective tables. For clarity in comparisons, we removed adolescents from the sample who reported that they attended some form of schooling that was different from in-person, hybrid, or virtual (e.g., home schooling; *n* = 79). Thus, for the following analyses, our sample size is 1177 adolescents.

### School satisfaction and academic success

To test H1 and H5, a MANCOVA was conducted with the four dependent measures of school satisfaction: feelings about school, perceptions of the meaningfulness of schoolwork, perceptions of coursework interest, and satisfaction with school. The multivariate test showed a main effect of school context, Wilks’ λ = .97, *p* < .0001, ηp^2^ = .015, and gender, Wilks’ λ = .98, *p* = .014, ηp^2^ = .008, but the interaction was not significant, Wilks’ λ = .99, *p* = .392, ηp^2^ = .004.

#### School context main effect on school satisfaction

The between-subjects effects also showed a main effect of school context on each of the four indicators of school satisfaction, providing support for H1. Those who attended school virtually scored significantly lower on feelings about school relative to those who attended in-person (*p* < .0001) and in a hybrid format (*p* = .029). Those attending virtually also scored significantly lower on perceptions about the meaningfulness of schoolwork (*p* = .015) and perceptions of coursework interest (*p* < .0001) relative to those who attended school in-person. For school satisfaction, those who attended school virtually scored significantly lower relative to those who attended in-person (*p* < .0001) and in a hybrid format (*p* = .035; see Table 2).

**Table 2:** School satisfaction and social connection by school context and gender identity.

	School Context			Gender Identity		
	In-Person	Hybrid	Virtual	Female	Male	TGNC
School Satisfaction & Success						
Feelings about school	2.96_b_	2.84_b_	2.41_a_	2.84_b_	2.91_b_	2.46_a_
Meaningfulness of schoolwork	2.85_b_	2.71_ab_	2.54_a_	2.86_b_	2.75_ab_	2.50_a_
Coursework interest	2.61_b_	2.37 _ab_	2.20_a_	2.50_b_	2.51_b_	2.15_a_
School satisfaction	3.13_b_	2.90_b_	2.48_a_	2.95_b_	2.97_b_	2.59_a_
Drop in Grades	.24_a_	.41_a_	1.23_b_	.37_a_	.44_a_	1.07_b_
Social Connection & Inclusion						
Satisfaction with peer connections	2.77_b_	2.58_ab_	2.31_a_	2.55_b_	2.78_c_	2.32_a_
Sense of inclusion in a social group	2.83_b_	2.54_ab_	2.49_a_	2.65_b_	2.94_c_	2.27_a_

Means with different subscripts indicate significant mean differences at *p* < .05. Comparisons are only within the three school context variables or within the three gender identity variables, as there were no significant school context by gender interactions. Exact *p*-values are reported in the text. Results after controlling for age, parent education, participant ethnicity, and virtual school duration.

#### Gender main effect on school satisfaction

The between-subjects effects showed a main effect of gender on each of the four indicators of school satisfaction, providing support for H5. TGNC youth scored significantly lower on feelings about school (compared to females: *p =* .009; males: *p* = .003), perceptions of the meaningfulness of schoolwork (females: *p* = .010; males = n.s.), perceptions of coursework interest (females: *p =* .014; males: *p* = .015), and lower levels of school satisfaction than their peers (females: *p =* .019; males: *p* = .020; see Table 2).

#### Academic success

We next conducted an ANCOVA with grade difference as the dependent variable. The analysis showed main effect of school context (*F* (2, 1177) = 11.41, *p* < .0001) and a main effect of gender (*F* (2, 1177) = 4.69, *p* = .009), but no significant interaction (*F* (4, 1177) = 1.58, *p* = .178). Bonferroni post-hoc analyses indicated that participants who were in virtual schooling reported a greater drop in grades relative to those attending in-person (*p* < .0001) or in hybrid format (*p* = .014). Post-hoc tests showed that TGNC youth had a greater drop in grades from the 2019–2020 school year to the 2020–2021 school year when compared to females (*p* = .007) and males (*p* = .026; see Table 2). This provides additional support for H1 and H5.

### Social connection and group inclusion

To test H2 and H6, we conducted a MANCOVA with our two variables of social connection as dependent variables: satisfaction with peer connection and sense of inclusion in a social group. The multivariate test showed a main effect of school context, Wilks’ λ = .97, *p* < .0001, ηp^2^ = .015, and gender, Wilks’ λ = .96, *p* < .0001, ηp^2^ = .019, but the interaction was not significant, Wilks’ λ = .99, *p* = .277, ηp^2^ = .004.

#### School context main effect on social connection

The between-subjects effects also showed a main effect of school context on satisfaction with peer connection and sense of inclusion in a social group, providing support for H2. Those who attended school virtually scored significantly lower on satisfaction with peer connection relative to those who attended in-person (*p* < .0001). For sense of inclusion in a social group, those who attended school virtually scored significantly lower relative to those who attended in-person (*p* = .016; see Table 2).

#### Gender main effect on social connection

The between-subjects effects showed a main effect of gender on satisfaction with peer connection and sense of inclusion in a social group, providing support for H6. For satisfaction with peer connection, TGNC youth scored significantly lower in comparison to both females (*p* = .041) and males (*p* < .0001). Further, males scored significantly higher than females (*p* < .0001). For sense of inclusion in a social group, TGNC youth scored significantly lower in comparison to both females (*p* = .013) and males (*p* < .0001). Further, males scored significantly higher than females (*p* = .001; see Table 2).

### Mental health

To test H3 and H7, we conducted a MANCOVA with our four variables of mental health as dependent variables: loneliness, anxiety, social anxiety, and depression. The multivariate test showed a main effect of school context, Wilks’ λ = .98, *p* = .008, ηp^2^ = .009, and a main effect of gender, Wilks’ λ = .89, *p* < .0001, ηp^2^ = .055, but the interaction was not significant, Wilks’ λ = .98, *p* = .326, ηp^2^ = .004.

#### School context main effect on mental health

The between-subjects effects showed a main effect of gender on depression and general anxiety, providing partial support for H3. Those who attended school virtually reported higher depression (*p* < .0001) and general anxiety (*p* = .017) relative to those who attended in-person (see Table 3).

**Table 3:** Mental health by school context and gender.

	School Context			Gender Identity		
	In-Person	Hybrid	Virtual	Female	Male	TGNC
Loneliness	2.89_a_	2.94_a_	3.10_a_	2.98_b_	2.71_a_	3.25_c_
Social anxiety	3.25_a_	3.27_a_	3.39_a_	3.37_b_	3.01_a_	3.53_b_
Depression	2.25_a_	2.43_ab_	2.63_b_	2.47_b_	2.00_a_	2.84_c_
Anxiety	2.93_a_	3.13_ab_	3.24_b_	3.15_b_	2.50_a_	3.64_c_

Means with different subscripts indicate significant mean differences at *p* < .05. Comparisons are only within the three school context variables or within the three gender identity variables, as there were no significant school context by gender interactions. Exact *p*-values are reported in the text. Results after controlling for age, parent education, participant ethnicity, and virtual school duration.

#### Gender main effect on mental health

The between-subjects effects showed a main effect of gender on loneliness, social anxiety, depression, and anxiety, providing support for H7. TGNC youth were higher on loneliness (compared to females: *p* = .026; males: *p* < .0001), social anxiety (females: n.s.; males: *p* < .0001), depression (females: *p* = .001; males: *p* < .0001), and general anxiety compared to their peers (females: *p* < .0001; males: *p* < .0001). Additionally, females were higher in loneliness, social anxiety, depression, and anxiety than males (all comparisons *p* < .0001; see Table 3).

### Media use

To test H4 and H8, we conducted a MANCOVA with four dependent measures of media use: social media use for social connection, problematic social media use, video gaming for social connection, and problematic video gaming. The multivariate test showed a main effect of school context, Wilks’ λ = .97, *p* < .0001, ηp^2^ = .017, and a main effect of gender, Wilks’ λ = .80, *p* < .0001, ηp^2^ = .106, but the interaction was not significant, Wilks’ λ = .98, *p* = .332, ηp^2^ = .005.

#### School context main effect on media use

The between-subjects effects also showed a main effect of school context on each of the four indicators of media use, providing partial support for H4, because some differences were opposite of what we hypothesized. Those who attended a hybrid model scored significantly higher on social media use for social connection relative to those who attended in-person (*p* = .006). Those attending school virtually scored significantly higher on both problematic social media use (*p* = .018) and problematic video gaming (*p* < .0001), both relative to those attending school in-person. Finally, those attending school virtually also scored higher on video gaming for social connection, relative to those attending in-person (*p* = .001; see Table 4).

**Table 4:** Media use by school context and gender.

	School Context			Gender Identity		
	In-Person	Hybrid	Virtual	Female	Male	TGNC
Social Media Use						
For Social Connection	2.85_a_	3.27_b_	2.98_ab_	2.97_a_	2.85_a_	3.29_b_
Problematic Use	2.30_a_	2.48_ab_	2.56_b_	2.55_b_	2.12_a_	2.67_b_
Video Game Use						
For Social Connection	2.61_a_	2.85_ab_	3.09_b_	2.28_a_	3.31_c_	2.96_b_
Problematic Use	1.85_a_	1.95_ab_	2.22_b_	1.71_a_	2.16_b_	2.16_b_

Means with different subscripts indicate significant mean differences at *p* < .05. Comparisons are only within the three school context variables or within the three gender identity variables, as there were no significant school context by gender interactions. Exact *p*-values are reported in the text. Results after controlling for age, parent education, participant ethnicity, and virtual school duration.

#### Gender main effect on media use

The between-subjects effects showed a main effect of gender on all four indicators of media use, providing partial support for H8, because some differences were opposite of what we hypothesized. TGNC youth scored significantly lower on social media use for social connection relative to males (*p* = .010). Males scored significantly higher on problematic social media use relative to females (*p* < .0001) and TGNC youth (*p* < .0001). Both males (*p* < .0001) and TGNC youth (*p* < .0001) scored higher on video gaming for social connection relative to females, but both males (*p* < .0001) and TGNC youth (*p* < .0001) also scored higher on problematic video gaming, relative to females, (see Table 4).

### Interactions

RQ1 asked if there would be a school context by gender interaction on any of our outcome variables. Interestingly, although each analysis showed a main effect of school context and gender, there were no significant interactions, providing answer to RQ1.

## Discussion

These analyses show a remarkably consistent and unambiguous pattern of results, supporting the contentions of the diathesis-stress model that certain individual differences may make individuals more susceptible to negative outcomes following the experience of stress, in this case stress due to the COVID-19 pandemic. Indeed, it is clear from this study that certain individuals ended the 2020–2021 school year facing more adversity than others. TGNC adolescents and adolescents in virtual learning both reported a more significant drop in academic success compared to the previous school year as well as less satisfaction with school. They reported feeling less socially connected and felt less inclusion with a peer group, while also reporting higher rates of mental health problems. Importantly, while adolescent youth are adept and frequent media users, and report using media for social purposes, in this instance in which so much of their in-person social connection was lost, social media and gaming do not appear able to provide a protective mechanism strong enough to compensate for that loss. In fact, problematic media use (both social media and video gaming) was highest by those in virtual learning contexts. It is critical that we recognize that all youth are not returning to school for the 2021–2022 school year with the same consequences of the COVID-19 pandemic, as a function of certain unique vulnerabilities, and that resources need to be in place to specifically support TGNC youth and those who were studying virtually at the end of last year, particularly around social connection and mental health.

### TGNC youth

A critical finding from this study was that TGNC youth reported less satisfaction with school, lower levels of social connections and inclusion with a peer group, a larger drop in overall grades, and more mental health problems (loneliness, depression, anxiety, and social anxiety) compared to their male or female peers, regardless of school context. This finding is consistent with previous work that demonstrates TGNC adolescents tend to struggle socially, academically, and in terms of mental health compared to their male or female peers [22, 26, 41, 42]. This finding is troubling for many reasons. First, this study demonstrates that one year after the start of the COVID-19 pandemic, TGNC youth were very much still struggling with school, social connectedness, and mental health at rates higher than their male or female peers, regardless of how they were attending school. For TGNC youth, removing the in-person school context did not seem to serve as a protective factor, minimizing the social and mental health consequences that this group tends to face. It is possible that virtual schooling cut down on the potential for in-person bullying, while simultaneously raising the potential for cyberbullying. Further, given that research suggests that TGNC youth already struggle to feel socially connected [5, 22, 25] virtual schooling may only have exacerbated this issue. The question thus remains: how then do we create spaces that are supportive and safe for TGNC youth to improve these outcomes?

#### School context

A second key finding from this study was that generally, adolescents attending school in-person reported more satisfaction with school, a greater sense of social connection and inclusion with a peer group, less of a drop in overall grades, and fewer mental health problems (depression and anxiety) compared to their peers who were attending school virtually. Those attending in a hybrid model generally scored between those in virtual schooling and those in-person, with few significant differences between the two. Importantly, these findings occurred when controlling for the length of time adolescents reported spending in virtual schooling, suggesting that any length of virtual schooling was associated with multiple negative outcomes. Further, these findings suggest that attending in-school at the end of last year seemed to offer some protective mechanism to youth to lessen the consequences of the stress associated with COVID pandemic in which other opportunities for socializing with friends was limited. In-person school context became a critical social lifeline for adolescent youth, and without that connection there was a clear consequence on social satisfaction, school engagement and success, and even mental health. This is consistent with past research on the role of social connection during adolescence [43–45] and extends it to show the role of in-person schooling in providing these key social inputs. This means that those who are returning to school in fall 2021 after learning virtually last year are potentially entering more at risk of social disconnectedness and poorer mental health than their peers, and will need supports in place to help them recover from the challenges faced last year, particularly the unique challenges of attending school virtually.

#### Media use

The need to socially distance due to COVID-19 resulted in many people around the world utilizing more media and in new and novel ways than ever before. Media use was undeniably higher and that was no different for adolescents who were already frequent media users [46, 47]. In this study we found that media use differed somewhat by both school context and gender identity. Although adolescents attending school virtually did report higher use of video gaming for social connection, they did not report higher use of social media for social connection. Interestingly, students who attended school in a hybrid model reported more use of social media for social connection than those attending school in-person. This may suggest that social media did not, or could not, replace the social connection that was lacking from the virtual learning context, but rather social media served as a tool to connect and extend the socialization that was cut short with hybrid in-person learning. Other recent reports indicated that about 20% of adolescents and young adults felt social media was “very important” to helping them feel less alone and getting support from others during the COVID-19 pandemic [47], but in our study we found that students who were learning virtually reported higher levels of problematic media use (both social media and video gaming). It thus appears that adolescents in virtual school environments were not able to effectively use media to help grow or maintain social connections, but rather, used media in a less adaptive way, which can itself contribute to mental health concerns [48]. Future research must examine whether this problematic media use continues as the majority of youth transition back to in-person learning.

Given the potential for online communication tools to allow for more selective self-presentation [33, 34] it was not surprising that TGNC youth reported higher levels of social media use for social connection compared to their male or female peers. This suggests that TGNC youth may be using social media to connect with peers in ways that feel more comfortable and safer for them. However, TGNC and female adolescents also reported higher levels of problematic social media use compared to male adolescents, suggesting that adolescent youth can perceive their use of media both positively (for social connection) and negatively (problematic use) simultaneously.

### Practical implications

The cross-sectional data presented here are a descriptive analysis of adolescent self-reports collected approximately one year into a global pandemic that resulted in quarantines, school closures, and a fewer social opportunities for most adolescent youth. Consistent with the predictions of the diathesis-stress model, the data provide a consistent picture that certain youth were more at risk at this moment in time as a function of unique susceptibilities, particularly youth who were learning remotely and those who identified as TGNC. These data have implications for government, policy makers, school administrators, parents, and adolescents. First, adolescence is a period in which many common psychopathologies tend to form [49]. Initial data show that adolescents reported increased symptoms of depression and anxiety, and a decrease in life satisfaction two months after the start of the COVID-19 pandemic began [9]. Therefore, local and federal governments need to support mental health care for adolescents, particularly TGNC youth and those returning to in-school learning from virtual learning contexts, as they appear to be more at-risk relative to their peers. Similarly, schools and parents need to support youth in the return to social situations and in-school learning in a way that recognizes the potential challenges these youth may be bringing to the new school year as a function of the past 18 months of COVID-19 precautions and potentially a full year of virtual learning. These needs likely will be beyond that of just academic success, including the social, emotional, and mental health needs of youth. Finally, for those working with TGNC youth, programs need to be developed to support their social and mental health by creating safe and welcoming spaces for students to learn, regardless of school context.

### Strengths and limitations

This study provides novel, large-scale data about adolescent social connection, school satisfaction, academic success, mental health, and media use at a unique period in history, during the COVID-19 pandemic that closed many schools and significantly limited social connection due to health precautions. This study is not without limitations, however. As these data are cross-sectional, this study only documents one moment in time and is entirely self-report by adolescent volunteers. Moreover, this study was conducted online, yet again forcing youth to utilize technology without the context of social connection to answer the questions. Further, although the sample is diverse, it is not nationally-representative, and therefore, it is not clear how these findings might generalize to all American adolescents.

In conclusion, this large survey of adolescents in the spring of 2021 documents the critical consequences for adolescents during the COVID-19 pandemic, particularly for those who were still in virtual schooling and TGNC youth. Moreover, it appears that their ability to utilize media as a protective mechanism to support social connection was not sufficient. In fact, many adolescents reported high levels of problematic social media and video game use, particularly if they were in virtual schooling context. The data from this study can and should be used to support adolescents in rebuilding social connection post-pandemic, and can encourage policy makers to recognize the critical need for social connections for all adolescents, but with careful consideration for how to support TGNC youth to ensure that they are feeling safe and socially connected in their schooling environment, regardless learning context.
